# Efficient sequence alignment against millions of prokaryotic genomes with LexicMap

**DOI:** 10.1038/s41587-025-02812-8

**Published:** 2025-09-10

**Authors:** Wei Shen, John A. Lees, Zamin Iqbal

**Affiliations:** 1 Department of Infectious Diseases, Key Laboratory of Molecular Biology for Infectious Diseases (Ministry of Education), Institute for Viral Hepatitis, The Second Affiliated Hospital, Chongqing Medical University, Chongqing, China; 2 European Molecular Biology Laboratory, European Bioinformatics Institute, Hinxton, UK; 3 Milner Centre for Evolution, University of Bath, Bath, UK

**Keywords:** Genome informatics, Bacterial genomics, Computational models, Genetic databases, Software

## Abstract

The size of microbial sequence databases continues to grow beyond the abilities of existing alignment tools. We introduce LexicMap, a nucleotide sequence alignment tool for efficiently querying moderate-length sequences (>250 bp) such as a gene, plasmid or long read against up to millions of prokaryotic genomes. We construct a small set of probe *k*-mers, which are selected to efficiently sample the entire database to be indexed such that every 250-bp window of each database genome contains multiple seed *k*-mers, each with a shared prefix with one of the probes. Storing these seeds in a hierarchical index enables fast and low-memory alignment. We benchmark both accuracy and potential to scale to databases of millions of bacterial genomes, showing that LexicMap achieves comparable accuracy to state-of-the-art methods but with greater speed and lower memory use. Our method supports querying at scale and within minutes, which will be useful for many biological applications across epidemiology, ecology and evolution.

## Abstract (web summary)

LexicMap uses a fixed set of probes to efficiently query gene sequences for fast and low-memory alignment.

## Main

Alignment of sequences to a single reference genome is a well-studied problem^1–4^. For specified gap and mismatch costs, dynamic programming is guaranteed to obtain the optimal solution^5–7^ but processing time scales as a product of the reference genome size and the query size. This is too slow in practice; thus, the challenge is to find faster shortcuts that ideally are still guaranteed to give the optimal solution. Rapid progress has recently been made on this front^8–12^.

The problem we set out to address is that of aligning to a database of genomes from across the bacterial phylogeny, as popularized by the basic local alignment search tool (BLAST)^1^. As the amount of publicly available bacterial sequencing data has grown over recent years, the proportion of bacterial genomes that web BLAST is able to search has dropped exponentially^13^. The primary use cases of alignment to either all bacterial genomes or a representative set are determining where a specific sequence has been seen before, finding the host range of a mobile element or gene or locating probable orthologs for further analysis. More broadly, the power to perform this alignment against all prior prokaryotic sequences would enable a wide range of specific analyses, just as BLAST has achieved with smaller datasets. Some concrete examples were seen in how approximate (*k*-mer-based) matching to the 661k dataset^14^ was used to search for plasmids^15–18^, adhesins^19^, diversity of vaccine targets^20^, mutations of interest^21^ and phages^15^.

This use case differs from mapping to one reference in two regards. Firstly, the scale of the intended database is larger in terms of number and diversity. For example, the GTDB r214 representative set^22^, one genome for each of ~85,000 bacterial species, contains 242 billion unique 31-mers—about 65,000 times more than in one genome. The diversity of gene content of bacteria is very large because many have ‘open’ pangenomes^23,24^; hence, so every new genome adds novel sequence content. Other natural databases to query are larger but heavily oversample pathogen species; in combination, GenBank^25^ and RefSeq^26^ contain 2.3 million genomes and AllTheBacteria^27^ contains 1.8 million high-quality genomes. Thus, there is a computational challenge to index these large databases. Secondly, if mapping to one reference, one tries to find the single most likely source of a query but rarely reports all alignments; by contrast, in the BLAST search use case, a sequence could truly come from multiple genomes and all alignments would potentially be wanted by the user.

There are a number of high-performance tools that are good options for large-scale alignment. MMseqs2 (ref. ^28^) supports sensitive and scalable search of nucleotide sequences by searching translated nucleotide databases using a translated nucleotide query. Minimap2 (ref. ^4^), a long-read alignment tool mainly designed for a single, large reference genome, can also be used for alignment against the large scale of microbial genomes, as it can partition input sequences and sequentially index and search each partition. Two tools have demonstrated the ability to scale to huge databases, albeit each with a caveat. First, Phylign^13^ compresses genomes by leveraging phylogenetic information and then, given a query, uses a *k*-mer-based method COBS^29^ to prefilter genomes before performing base-level alignment with Minimap2. However, prefiltering on the basis of matching 31-mers is only effective for highly similar sequences. As the divergence of the query increases beyond 10%, it becomes very likely to fail the prefilter^30^. It is essential for most useful searches to avoid this limitation. Second, it was shown that, by restricting to the 179/11,264 species in AllTheBacteria^27^ that have >200 genomes (which is 94% of the data), a new BWT implementation, Ropebwt3 (ref. ^31^), can leverage the within-species redundancy and compress the data from ~2.8 TB (gzip-compressed) to just 27.6 GB. However, it is also important to be able to align to all the species in the dataset.

We need to be able to find anchoring matches shared between a query and a genome, with which we can do straightforward alignment. We create a relatively small set of probes (20,000 *k*-mers, much smaller than the 59 billion *k*-mers in AllTheBacteria or 292 billion *k*-mers in the GTDB complete dataset) that ‘cover’ the genomes in the database such that every 250-bp window contains several (median 5) *k*-mers, each with a 7-bp prefix match with one of our probes. Combining this core idea with a range of computational innovations, we develop a standalone alignment tool, LexicMap, which is able to align a gene to millions of genomes in minutes.

## Results

### Accurate seeding algorithm

**Fig. 1:**
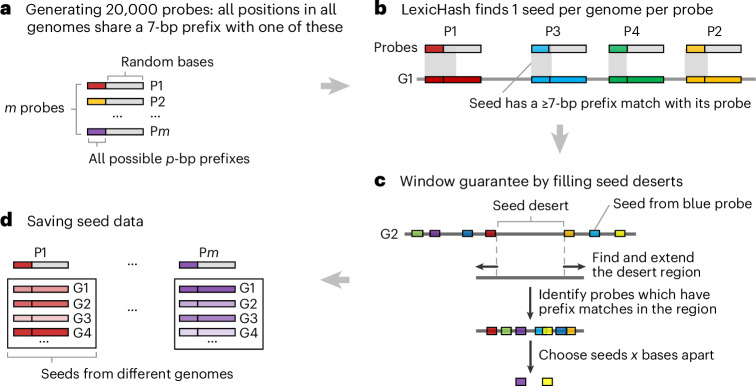
Seeding scheme of LexicMap for reference database. **a**, A fixed set of 20,000 31-mers (called probes) are generated, ensuring that their prefixes include every possible 7-mer. Seeds, each prefix matching one of these, will be found distributed across all database genomes and chosen in such a way as to have a window guarantee. **b**, LexicHash creates one hash function per probe and, when applied to a genome, it finds the *k*-mer with the longest prefix match, which is then stored as a seed. **c**, Each genome is scanned to find seed deserts (regions longer than 100 bp with no seed); every *k*-mer within this region has a 7-mer prefix match with at least one probe (because the probes cover all possible 7-mers); hence, seeds can be chosen with spacing of *x* bp (50 by default). **d**, Seeds are stored in a hierarchical index. In fact, although not shown here for simplicity, the number of seeds is doubled to support both prefix and suffix matching (details in Methods).

In LexicMap, we first reimplement the sequence sketching method LexicHash^32^, which supports variable-length substring matches (prefix matching) rather than fixed-length *k*-mers, and use this to compute alignment seeds. We outline the approach here and give full details in Methods. First, 20,000 31-mers (called ‘probes’) are generated (Fig. 1a), which can ‘capture’ any DNA sequence by prefix matching, as the probes contain all possible 7-bp prefixes. Then, for every reference genome, each probe captures one *k*-mer across the genome as a seed; this is conducted using the LexicHash, which chooses the *k*-mer that shares the longest prefix with the probe (Fig. 1b and Supplementary Fig. 1). The number 20,000 above was chosen as a tradeoff among index size, alignment accuracy and performance, as detailed below (Supplementary Tables 1 and 2 and Supplementary Fig. 2).

However, because the captured *k*-mers (seeds) are randomly distributed across a genome, the distances between seeds vary and there is initially no distance guarantee for successive seeds (Supplementary Fig. 3a). As a result, some genome regions might not be covered with any seed, creating ‘seed deserts’ or ‘sketching deserts’ (ref. ^33^), where sequences homologous to these regions could fail to align. Generally, seed desert sizes and numbers increase with larger genome sizes (Supplementary Fig. 3a). To address this issue, a second round of seed capture was performed for each seed desert region longer than 100 bp. New seeds spaced about 50 bp apart are added to the seed list of the corresponding probe (Fig. 1c,d). After filling these seed deserts, a seed distance of 100 bp is guaranteed (Supplementary Fig. 3b) for non-low-complexity regions, ensuring that all 250-bp sliding windows contain a minimum of two seeds, with a median of five in practice (Supplementary Fig. 3c). Additionally, the seed number of a genome may in practice be lower or higher than the number of probes; the seed number is generally linearly correlated with genome size (Supplementary Fig. 4). Lastly, by allowing variable-length prefix matches, we greatly increase sensitivity compared with a *k*-mer exact-matching approach but the method remains vulnerable to variation within the prefix region; therefore, we extended LexicMap to additionally support suffix matching (Methods).

### Scalable indexing strategies

To scale to millions of prokaryotic genomes, input genomes are indexed in batches to limit memory consumption, with all batches merged at the end (Supplementary Fig. 5a). Within each batch, multiple sequences (contigs or scaffolds) of a genome are concatenated with 1-kb intervals of Ns to reduce the sequence scale for indexing. Original coordinates and sequence identifiers are restored after sequence alignment. The complete genome or the concatenated contigs are then used to compute seeds using the generated probes as described above, with intervals and gap regions skipped. Genome sequences are saved in a bit-packed format along with genome information for fast random subsequence extraction. After indexing all reference genomes, each probe captures up to millions of *k*-mers, including position information (genome ID, coordinate and strand). A scalable hierarchical index compresses and stores seed data for all probes (Methods and Supplementary Fig. 5a) and supports fast, low-memory variable-length seed matching, including both prefix and suffix matching (Methods and Supplementary Fig. 5b).

### Efficient variable-length seed matching and alignment

**Fig. 2:**
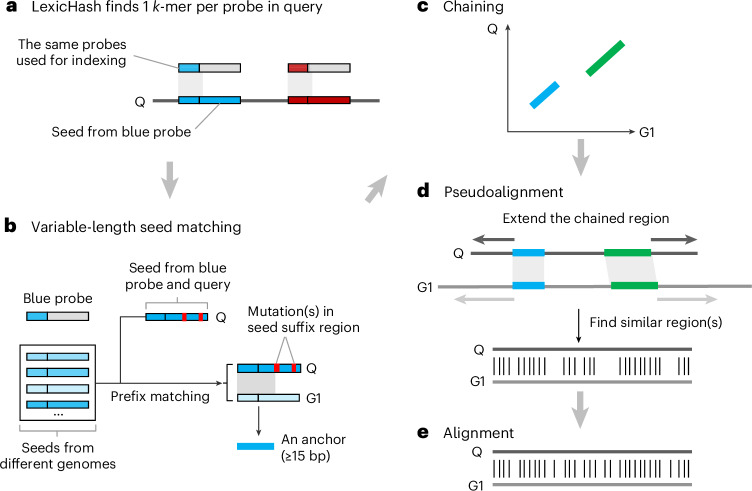
LexicMap alignment workflow. **a**, The same LexicHash hash functions (one per probe) used in the indexing step are used here, applied to the query to capture one prefix-matching *k*-mer per probe. **b**, For each probe, the seed data are scanned to find prefix or suffix matches ≥ 15 bp. The common prefix or suffix constitutes an anchor. **c**, The variable-length anchors are chained using a modified version of the Minimap2 algorithm. **d**,**e**, Fast pseudoalignment (**d**) is followed by base-level alignment (**e**) using the wavefront alignment algorithm. Note that, in **b**, only prefix matching is illustrated, whereas suffix matching is not shown for simplicity.

In the searching step, probes from the LexicMap index are used to capture *k*-mers from the query sequence (Fig. 2a). Each captured *k*-mer is then searched in the seed data of the corresponding probe to identify seeds that share prefixes or suffixes of at least 15 bp (chosen as a tradeoff between alignment accuracy and efficiency; Supplementary Table 3 and Supplementary Fig. 6), using a fast and low-memory approach (Methods, Fig. 2b and Supplementary Fig. 5b). The common prefix or suffix of the query and target seed, along with the position information, constitutes an anchor. These anchors are grouped by genome ID before chaining. The minimum anchor length of 15 bp ensures search sensitivity, while longer anchors (up to 31 bp) provide higher specificity. Unlike minimizer-based methods such as Minimap2, which use fixed-length anchors with a small window guarantee between anchors, LexicMap uses variable-length anchors and does not guarantee a fixed window size between anchors. Consequently, the chaining function (function 1 in Methods) assigns more weight to longer anchors and does not consider anchor distance (Methods). Next, a pseudoalignment is performed to identify similar regions from the extended chained regions (Fig. 2d). Finally, the wavefront alignment algorithm is used for base-level alignment (Fig. 2e). LexicMap’s default output is a tab-delimited table providing alignment details (Supplementary Table 4) for filtration or further analysis and also supports an intuitive BLAST-style pairwise alignment format (Supplementary Fig. 7).

### Robustness to sequence divergence

LexicMap supports variable-length seed matches through prefix and suffix matching, allowing greater tolerance to mutations compared with fixed-length seeding methods. To evaluate LexicMap’s robustness to sequence divergence, ten bacterial genomes from common species with sizes ranging from 2.1 to 6.3 Mb (Supplementary Table 5) were used to simulate queries of varying lengths and similarities by introducing single-nucleotide polymorphisms (SNPs) and indels with Badread^34^ (Methods). BLASTn (with word sizes of both the default 28 and 15), MMseqs2, Minimap2 and Ropebwt3 were compared with LexicMap. Additionally, COBS was compared with a high-sensitivity setting (minimum fraction of aligned *k*-mers: 0.33), as it is used in the prefilter step of Phylign.

**Fig. 3:**
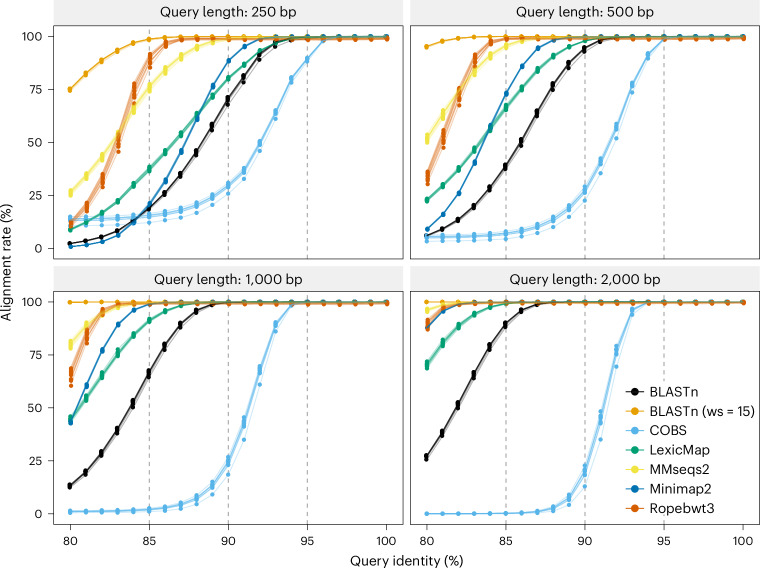
Robustness of aligners to sequence divergence. This is measured by simulating 250-bp, 500-bp, 1,000-bp and 2,000-bp reads with coverage of 30× from ten bacterial genomes, adding mutations to achieve sequence divergence between 0% and 20% and then aligning back to the source genome. COBS is a *k*-mer index; thus, we show what proportion of the reads were detected as being present in the source genome (rather than being aligned). This falls off very rapidly as similarity drops because each base disagreement loses an entire 31-mer. For the other tools, we measure the proportion of reads that are correctly aligned back to the source genome. BLASTn (ws = 15) represents BLASTn with a word size of 15. BLASTn (with no brackets) refers to the default setting of BLASTn, with word size of 28. All data are available in Supplementary Table 6.

**Extended Data Fig. 1:**
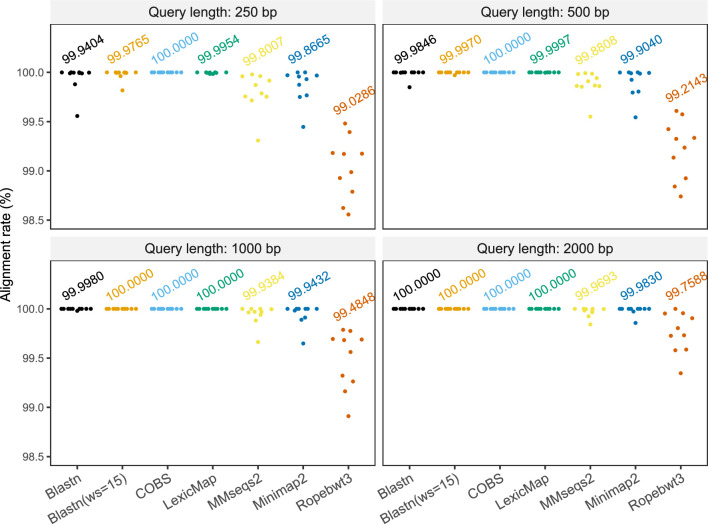
Alignment rates of simulated mutation-free queries. Queries with 100% identity (a subset of the experiment shown in Fig.3) are aligned; the proportion of these which are aligned to the right place are shown here. Text labels are the mean alignment rates. All data are available in Supplementary Table 6.

Generally, as query identity increased, alignment rates of all tools improved, reaching nearly 100% for query identities ≥ 95% when query length was ≥500 bp (Fig. 3 and Supplementary Table 6). For queries of 1,000 bp and 2,000 bp, BLASTn with a word size of 15 bp consistently achieved the highest alignment rates at lower query identities, followed by MMseqs2, Ropebwt3, Minimap2, LexicMap and BLASTn with the default word size of 28 bp. COBS showed a steeper dropoff in alignment rates at query identities below 95%, which is expected given that it relies on comparing fractions of matched *k*-mers (*k* = 31). For 250-bp and 500-bp queries, LexicMap outperformed default BLASTn at query identities below 93% and 92% and surpassed Minimap2 at query identities below 88 and 83%, respectively. The performance for mutation-free queries is shown in Extended Data Fig. 1.

### Scalability to 1 million genomes

To evaluate the scalability of the above sequence alignment and search tools to increasing prokaryotic genomes, we created seven genome sets at varying scales (ranging from 1 to 1 million genomes) by randomly selecting nonoverlapping prokaryotic genomes from GenBank and RefSeq databases. Next, we built an index per set with each tool and then performed searches using a query set containing a rare gene (*secY* from *Enterococcus faecalis*) and a 16S rRNA gene (*rrsB* from *Escherichia coli*) (Methods).

**Fig. 4:**
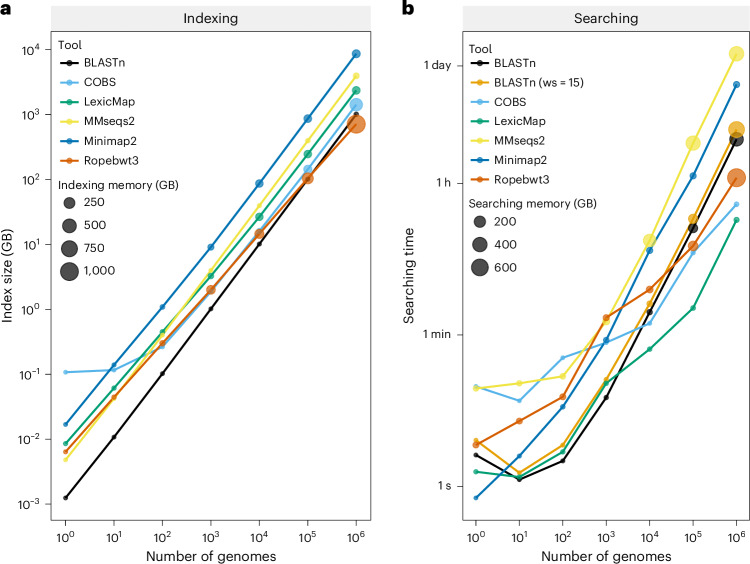
Scalability of sequence alignment and search tools. Benchmarking BLASTn, COBS, LexicMap, MMseqs2, Minimap2 and Ropebwt3 for both index construction and subsequent querying, using datasets ranging from 1 to 1 million genomes. **a**, Index size and memory requirements for index construction. **b**, Search (alignment for BLASTn, LexicMap, MMseqs2, Minimap2 and Ropebwt3 and search for COBS) time and memory use. The query set consists of a rare gene (*secY*) and a 16S rRNA gene (*rrsB*) sequence. All tools return all possible matches. Performance data are in Supplementary Tables 7 and 8.

For index building, generally, the index sizes of all tools were linearly correlated with the number of genomes (Fig. 4a). In terms of memory requirements to index 1 million genomes, Ropebwt3 required the most memory (1,013 GB), followed by COBS (382 GB), Minimap2 (85 GB), LexicMap (75 GB), MMseqs2 (20 GB) and BLASTn (2 GB). The indexing time of all tools varied (Supplementary Table 7), ranging from 2.6 h (MMseqs2) to 23.3 days (Ropebwt3). For databases larger than 10,000 genomes, LexicMap outperformed all other alignment tools in terms of alignment time and memory usage (Fig. 4b; note the log scale on *y* axis). For databases of 1 million genomes, LexicMap was three times faster than the second fastest alignment tool (Ropebwt3) while using only 1/115 of the memory (6.2 GB versus 717 GB); it was 89 times faster than MMseqs2 and 39 times faster than Minimap2.

### Indexing performance on large databases

We further evaluated the performance and accuracy of LexicMap in the three largest and most diverse datasets (Methods): the GTDB r214 complete dataset with 402,538 prokaryotic assemblies, the AllTheBacteria version 0.2 high-quality dataset with 1,858,610 bacterial assemblies and GenBank + RefSeq with 2,340,672 prokaryotic assemblies (downloaded on February 15, 2024). We benchmarked against BLASTn, Minimap2, MMseqs2 and Phylign. Ropebwt3 was excluded from this benchmark because it did not scale to this dataset (as outlined above) or return all alignments.

In terms of index sizes for AllTheBacteria, Phylign had the smallest size (Table 1), followed by BLASTn. LexicMap, MMseqs2 and Minimap2 had index sizes approximately 2.5, 4 and 8 times larger than BLASTn, respectively. For indexing time, MMseqs2 was the fastest, followed by BLASTn, Minimap2 and LexicMap. For indexing memory, BLASTn used the least memory, followed by MMseqs2, Minimap2 and LexicMap. LexicMap used almost twice as much memory for the GenBank + RefSeq dataset compared with the AllTheBacteria dataset, as the genomes in the former are more diverse.

**Table 1:** LexicMap indexing performance on three datasets

Dataset	Assemblies	Bases	Tool	Index size	Time	RAM
GTDB complete	402,538	1.5 Tbp	LexicMap	972 GB	9 h 39 min	71.6 GB
			BLASTn	387 GB	3 h 11 min	718 MB
			MMseqs2	1,555 GB	55 min	7.6 GB
			Minimap2	3,409 GB	6 h 51 min	69.7 GB
AllTheBacteria	1,858,610	7.5 Tbp	LexicMap	4,305 GB	40 h 22 min	97.7 GB
			BLASTn	1,932 GB	14 h 03 min	2.9 GB
			MMseqs2	7,551 GB	5 h 14 min	8.2 GB
			Minimap2	15,541 GB	32 h 08 min	76.5 GB
			Phylign	248 GB	-	-
GenBank+RefSeq	2,340,672	9.2 Tbp	LexicMap	5,455 GB	58 h 52 min	174.4 GB
			BLASTn	2,368 GB	14 h 04 min	4.3 GB
			MMseqs2	9,259 GB	6 h 41 min	8.1 GB
			Minimap2	20,283 GB	42 h 30 min	76.2 GB

LexicMap built indices with the genome batch size of 5,000 for GTDB and 25,000 for the AllTheBacteria and GenBank + RefSeq datasets.

### Alignment accuracy and performance on large databases

Four different types of queries were used to evaluate alignment performance (Methods): (1) a comparatively rare gene (BLASTn returns 7,000 genome hits in the GTDB r214 complete dataset with 402,538 genomes)—*secY* from *E.* *faecalis*; (2) a 16S rRNA gene *rrsB* from *E.* *coli*; (3) a 53-kb plasmid; and (4) 1,033 different AMR genes (batch queries).

First, we aligned the queries with LexicMap, BLASTn, MMseqs2 and Minimap2 against the GTDB complete index. For clearer comparison, we divided all alignment results into three groups (high, medium and low similarity; Table 2). High-similarity alignments were long with high identity, low-similarity alignments were either short or highly diverged and medium-similarity alignments constituted the remainder (precise definitions in Methods).

**Table 2:** Alignment performance benchmarks on GTDB complete dataset

Query (length)	Tool	Hits (total)	Hits (high)	Hits (medium)	Hits (low)	Time	RAM
A rare gene 1,299 bp	LexicMap	6,255	2,311	46	3,898	30 s	2.1 GB
	BLASTn	7,121	2,311	47	4,763	2,171 s	351.2 GB
	BLASTn (ws = 15)	57,741	2,311	47	55,383	3,171 s	324.1 GB
	MMseqs2	67,537	2,304	54	65,179	26,174 s	400.7 GB
	Minimap2	2,312	2,312	0	0	17,208 s	20.2 GB
A 16S rRNA gene 1,542 bp	LexicMap	306,064	60,999	69,293	175,772	303 s	5.2 GB
	BLASTn	301,197	61,878	109,477	129,842	2,760 s	378.4 GB
	BLASTn (ws = 15)	301,197	61,878	109,477	129,842	3,291 s	378.4 GB
	MMseqs2	324,364	60,915	89,874	173,575	31,140 s	400.7 GB
	Minimap2	17,656	15,998	1,652	6	17,313 s	20.2 GB
A plasmid 52,830 bp	LexicMap	65,029	21	2,808	62,200	539 s	6.8 GB
	BLASTn	69,311	21	2,865	66,425	2,262 s	364.7 GB
	BLASTn (ws = 15)	91,847	21	2,865	88,961	3,082 s	142.8 GB
	MMseqs2	90,277	7	1,650	88,620	44,710 s	400.7 GB
	Minimap2	3,033	35	1,873	1,125	19,715 s	20.2 GB
1,033 AMR genes 1 kb (median)	LexicMap	4,665,317	1,123,251	776,153	2,765,913	8,620 s	10.7 GB
	BLASTn	5,357,772	1,150,407	772,858	3,434,507	4,686 s	442.1 GB
	BLASTn (ws = 15)	10,877,544	1,150,410	840,464	8,886,670	4,561 s	311.9 GB
	MMseqs2	10,137,345	1,148,942	808,177	8,180,226	184,470 s	406.9 GB
	Minimap2	2,078,490	943,516	39,529	815,445	38,058 s	20.2 GB

A high-similarity alignment has query coverage of ≥90% (for genes) or ≥70% (for plasmids) and identity of >90%. A low-similarity alignment has query coverage of <50% (genes) or <30% (plasmids) and identity of <80%. All the remaining alignments are classified as medium similarity. Hits stand for genome hits. Hits (high), hits (medium) and hits (low) denote the number of genomes with high-similarity, medium-similarity and low-similarity matches, respectively.

In short, all tools found very similar numbers of high-similarity alignments but LexicMap reported fewer low-similarity alignments; that is, it had lower sensitivity to highly diverged (identity<80%) or short fragmentary alignments. For the rare gene, all tools returned almost identical numbers of high-similarity alignments but MMseqs2 and BLASTn reported about ten times more low-similarity alignments than other tools. For the 16S rRNA gene, where we expected to find many alignments, LexicMap, BLASTn (both settings) and MMseqs2 reported ~61,000 high-similarity alignments, whereas Minimap2 only reported ~16,000. Counting all (high-similarity, medium-similarity and low-similarity) alignments, all tools except Minimap2 found around 300,000 alignments (MMseqs2 found the most at 324,000), whereas Minimap2 reported ~18,000. In contrast, the 53-kb plasmid, which is likely to present a different type of challenge, with many small fragmentary hits and some long hits with large deletions, revealed other differences between the tools. LexicMap and BLASTn (both settings) found 21 high-similarity matches and Minimap2 finds 35 but MMseqs2 founds only 7. However, BLASTn (ws = 15) and MMseqs2 found many more low-similarity alignments than other tools. Lastly, for the AMR genes, LexicMap, BLASTn (both settings) and MMseqs2 found around 1.1 million alignments, whereas Minimap2 found 943,000. However, BLASTn (ws = 15) and MMseqs2 again reported four times more low-similarity alignments.

In terms of speed, LexicMap was much faster than other tools for single queries, being 72, 9 and 4 times faster than the second fastest tool BLASTn, on the rare gene, 16S rRNA gene and plasmid respectively. Compared with MMseqs2, LexicMap was 872, 103 and 83 times faster for the same queries. For batch queries, LexicMap was 1.8 times slower than BLASTn. Regarding memory usage, LexicMap required less than 7 GB for single queries and 11 GB for the 1,033 AMR genes, whereas Minimap2 used 20.2 GB and BLASTn and MMseqs2 used more than 300 GB across all queries.

Next, we compared LexicMap to Phylign on the AllTheBacteria dataset, which contains 1,858,610 bacterial genomes, including some species that are highly oversampled. Here, BLASTn could not be run because of its requirement of more than 2,000 GB of memory. MMseqs2 was not included for its slow speed and Minimap2 was not included for its slow speed and lower sensitivity for medium-similarity and low-similarity matches. Across all queries, if including all (high-similarity, medium-similarity and low-similarity) hits, LexicMap returned more genome hits than Phylign; however, for high-similarity matches, the number of alignments was very similar (Extended Data Table 1). These observations are as expected given the effect of Phylign using a *k*-mer filter on diverged hits (Fig. 3). In terms of computation efficiency, for single queries, LexicMap took much less time than Phylign in both local and cluster mode (using up to 100 nodes) while also using much less memory. However, for batch querying, LexicMap was much slower than Phylign in local and cluster modes; however, in both cases, LexicMap returned more alignments.

Lastly, we tested LexicMap on the GenBank + Refseq dataset (234 million prokaryotic genomes), where it achieved similar performance to that on the AllTheBacteria dataset (Extended Data Table 2).

## Discussion

BLAST was not the first tool to enable DNA alignment against a database but its speed and accessibility revolutionized bioinformatics. However, since then, the National Center for Biotechnology Information BLAST has been querying an exponentially smaller fraction of public data^13^. The vast majority of the cellular tree of life consists of bacteria and archaea and we continue to expand the tree through metagenomic sequencing. Although the rate of discovery of new phyla has dropped, this is not the case for lower taxa^24^. Taken together with the prevalence of horizontal gene transfer in prokaryotes and a high number of mobile genetic elements and their cargo, the levels of diversity are extremely high and continue to grow. Furthermore, the amount of clinical sequencing and deposition in archives continues to grow our collection of pathogen sequence data, providing a year-by-year perspective on real-time evolution and horizontal gene transfer. Thus, now more than ever, the ability to align a query against a database of representative genomes or all genomes is vital to modern biology and public health. Developers of new antibiotics who find specific mutations that confer resistance should be able to find out whether those SNPs have been seen before, representing preexisting resistance^35^. Genomic epidemiologists should be able to query a drug-resistant plasmid from a hospital outbreak against recently sequenced genomes from across the world^15^ or track down any global samples containing outbreak informative SNPs^36^. Just as BLAST enabled hundreds of different studies that could not have been predicted at the time, re-enabling alignment against all bacteria will surely potentiate a wide range of new applications.

We introduced our solution to this problem here. LexicMap constructs a fixed set of 20,000 probes (*k*-mers) that are guaranteed to have multiple (around five in this study) prefix matches in every 250-bp window of every genome in the database. The *k*-mer with the best prefix match for each probe in each genome (called a seed) is stored in a hierarchical index, which can be used for alignment. This use of variable-length prefix and suffix matching enables sensitive nucleotide alignment for queries above 250 bp long (although this is a user-tunable threshold). Our results showed (Fig. 4) that LexicMap achieves a superior scalability to the other benchmarked alignment tools, with the fastest speed and the lowest memory usage, while maintaining a moderate index size and indexing efficiency. While achieving this scalability, LexicMap maintains a comparable sensitivity (meaning robustness to divergence of the query from the target) to state-of-the-art aligners.

LexicMap provides direct alignment without a lossy prefilter step. Additionally, all possible matches, including multiple copies of genes in a genome, are returned. The alignment is fast and memory efficient; moreover, unlike minimizer-based methods, seeds in LexicMap are interpretable and several utility commands are available to interpret probe (probe *k*-mers) and seed (seed sequences and positions) data. LexicMap is easy to install on multiple operating systems and can be used as a standalone tool without needing a workflow manager or compute cluster. LexicMap mainly supports small genomes including complete or partially assembled prokaryotic, viral and fungal genomes. The maximum supported sequence length is 268,435,456 bp (2^28^); thus, it can in principle also be applied to bigger genomes such as human genomes with a maximum chromosome size of 248 Mb.

In terms of limitations, LexicMap only supports queries longer than 250 bp. It achieves a low memory footprint by storing a very large index on disk (5.46 TB for 2.34 million prokaryotic assemblies in GenBank + RefSeq), although we do note that the corresponding index for MMseqs2 or Minimap2 would be larger. Nevertheless, it would be desirable in future to reduce the size of this index. Lastly, LexicMap is optimized for a small number of queries; improving batch searching speed is planned in the future.

LexicMap marks a step change in scalability, achieving low-memory queries of the global corpus of bacterial data in minutes. Coupled with its ease of installation and use, without the need for workflow managers or a cluster, it has the potential to enable a wide range of analyses from ecology, evolution and epidemiology.

## Methods

### Probe generation

Probes, also referred to as ‘masks’ in the LexicHash paper, consist of a fixed number (*m* = 20,000 by default) of *k*-mers (*k* ≤ 32, *k* = 31 by default), which capture DNA sequences by prefix matching. A probe consists of two parts—the *p*-bp prefix and the remaining bases. To enable probes to match all possible reference and query sequences by prefix matching, all permutations of *p*-mers are generated as the base prefix set. The length of the prefix (*p*) is calculated to ensure that the total number of possible prefixes (4^*p*^) does not exceed the total number of probes (*m*). For instance, when *m* = 20,000, *p* = 7. Then, the base prefixes are duplicated to reach the number of probes *m*. Next, the suffixes of probes are randomly generated. The *p*′-bp (*p*′ = *p* + 1) prefixes are required to be distinct to enable fast locating of the probe by the prefix of a *k*-mer through an array data structure. Lastly, these *m k*-mers serve as probes that can match all potential sequence regions in both reference genomes and query sequences.

### Seed computation

The *k*-mers are encoded as 64-bit unsigned integers, with a binary coding scheme of A = 00, C = 01, G = 10 and T = 11. Following the original implementation of LexicHash, the hash value between a probe and a *k*-mer is computed using a bitwise XOR operation. The value of this hash is that smaller hash values indicate longer common prefixes between the probe and the *k*-mer, whereas a hash value of zero means the probe and the *k*-mer are identical.

For each reference genome or query sequence, all *k*-mers from both forward and backward strands are compared with probes that share the same *p*-bp prefix. Each probe retains only the *k*-mer with the minimum hash value, which might in principle be located at more than one position. The *k*-mers of low-complexity are discarded by DUST algorithm^37^ with a loose score threshold of 50. A 64-bit integer encodes each position’s information, including the genome batch index (17 bits, as described below), genome index (17 bits), position (28 bits), strand (1 bit) and seed direction flag (1 bit, as described below). Ultimately, each probe captures one *k*-mer (as a seed) across the entire reference genome or query sequence, which shares the longest prefix with the probe.

However, because of the random distribution of captured *k*-mers (seeds), the distances between these seeds vary and there is no guarantee of consistent distance between successive seeds. Consequently, some regions of the sequence might remain uncovered by seeds, leading to what are known as ‘seed deserts’. These deserts are problematic because they can cause sequences homologous to the regions to fail to align. To address this issue, regions identified as seed deserts, which are longer than a certain threshold (100 bp by default), are extended by 1 kb both upstream and downstream. A second round of seed capture is then performed in these extended regions and new seeds are spaced about *x* bp (50 by default) apart within the region are added to the index of the corresponding probes. After filling these seed deserts, the total number of seeds may exceed the initial value of *m*.

### Indexing

The input of LexicMap is a list of microbial genomes, with the sequences of each genome stored in separate FASTA files. These files can be in plain or compressed formats such as gzip, xz, zstd or bzip2. Each file must have a distinct genome identifier in the file name. To limit memory consumption, genomes are indexed in batches and all batches are merged at the end (Supplementary Fig. 5a).

In each batch, genomes with any sequence larger than a genome size threshold (15 Mb by default), such as nonisolate assemblies, are skipped. On the other hand, if only the total length of sequences exceeds the threshold, the genomes are split into multiple chunks and alignments from these chunks will be merged in the searching step. Additionally, unwanted sequences within genomes, such as plasmids, can be optionally discarded using regular expressions to match sequence names. Multiple sequences (contigs or scaffolds) of a genome are concatenated with 1-kb intervals of Ns to reduce the scale of sequences to be indexed. The original coordinates will be restored after sequence alignment. The complete genome or the concatenated contigs are then used to compute seeds with generated probes, as described above. Before this, any degenerated bases are converted to their corresponding alphabet-first bases (for example, N is converted to A). The genome sequence is then saved in a bit-packed format (2 bits per base), along with associated genome information (genome ID, size and sequence IDs and lengths of all contigs) to enable fast random subsequence extraction. Simultaneously, seeds and their positional information are appended to their corresponding probes and saved as seed files. After processing all genomes in the batch, these seed files are merged using an external sorting method once all batches have been indexed.

### Seed data storage and variable-length seed matching

After indexing with all *n* reference genomes, each probe captures *n* or more seeds (*k*-mers, encoded as 64-bit integers), with each seed potentially having one or more positions (also represented as 64-bit integers), and there are *m* probes in total (20,000 by default). To scale to millions of prokaryotic genomes, the storage of seed data needs to be both compact and efficient for querying. Because the seeds of different probes are independent, the seed data are saved into *c* chunk files to enable parallel querying (Supplementary Fig. 5a). In each chunk file, the seed data of approximately *m*/*c* probes are simply concatenated. For each probe, all seeds are sorted in alphabetical order and the varint-GB^38^ algorithm is used to compress every two seeds along with their associated position counts. Because seeds captured by the same probe share common prefixes, the differences between two successive seeds are small, as are the position counts. As a result, two values can be stored using as little as 3 bytes instead of 16.

Unlike the approach in the LexicHash paper, where captured *k*-mers from each probe are stored in a prefix tree in main memory, here, they are alphabetically sorted and saved in a list-like structure within files. To enable efficient variable-length prefix matching of seeds, an index is created for each seed data file (Supplementary Fig. 5a,b). This index functions similarly to a table of contents in a dictionary, storing a list of marker *k*-mers along with their offsets (pages) in the seed data file. Each marker *k*-mer is the first one with a specific *p*″-bp subsequence (*p*″ = 6 by default) following the *p*-bp prefix (all seeds of a probe share the same *p*-bp prefix). For a query sequence, one *k*-mer is captured by each probe and searched within the corresponding probe’s seed data to return seeds that share a minimum length of prefix with the query *k*-mer. For example, searching with CATGCT for seeds (with *p* = 2, *p*″ = 1) that have at least 4 bp of common prefixes is equivalent to finding seeds in the range of CATGAA to CATGTT. The process starts by extracting the *p*″-bp subsequence from CATGAA (in this case, T) to locate the marker *k*-mer (for example, CATCAC) with the same *p*″-bp subsequence in the same region. The offset information (page) of this marker *k*-mer is then used as the starting point for scanning seeds within the *k*-mer range.

The index structure described above is extended to support the suffix matching of seeds. During the indexing phase, after a seed *k*-mer is saved into the seed data of its corresponding probe, the *k*-mer is reversed and added to the seed data of the probe that shares the longest prefix with the reversed *k*-mer. Additionally, the last bit of each position data is used as a seed direction flag, indicating that the seed *k*-mer is reversed. As a result, all seeds are doubled; there are ‘forward seeds’ for prefix matching and ‘reversed seeds’ for suffix matching, which is achieved through prefix matching of the reversed seeds. In the seed matching process, two rounds of matching are performed. The first round involves prefix matching (as described above) in the forward seed data. In the second round, the query *k*-mer is reversed and searched in the reversed seed data of the probe that shares the longest prefix with the reversed *k*-mer.

### Chaining

After searching all captured *k*-mers from a query in the seed data of all probes, seed pairs (anchors) with different matched prefix and suffix lengths *l* are returned. These anchors indicate matched regions between the query sequences [*y*, *y* + *l* − 1] on the strand ${s}_{y}$ and reference genome [*x*, *x* + *l* − 1] on strand ${s}_{x}$. First, the anchors are grouped by the genome IDs. Within each group, the anchors are sorted by the following criteria: (1) ascending order of start positions in the query sequence; (2) descending order of end positions in the query sequence; and (3) ascending order of start positions in the target genome. Only one anchor is kept for duplicated anchors and any inner anchors that are nested within other anchors are removed. Then, overlapped anchors with no gaps are merged to a longer one, whereas, for these with gaps, only the nonoverlapped part of the second anchor is used to compute the weight, as described below. Next, a chaining function modified from Minimap2 is applied to chain all possible colinear anchors:

1 $$f\left(i\right)=\max\left\{\mathop{\max}\limits_{\begin{array}{c}i> j\ge 1\\{x}_{i}-G < {x}_{j}\le{x}_{i}\\{y}_{i}-G <{y}_{j}\le {y}_{i}\\ {d}_{{ji}}\le D\end{array}}\left\{\;f\left(j\right)+{w}_{i}-\gamma \left({g}_{{ji}}\right)\right\},{w}_{i}\right\}$$

Here, $f\left(i\right)$ and $f\left(j\right)$ are scores for anchors ${a}_{i}$ and ${a}_{j}$, respectively. The anchor weight ${w}_{i}=0.1\times {l}_{i}^{2}$, where ${l}_{i}$ is the length of the anchor ${a}_{i}$ and $P\le {l}_{i}\le k$, with *P* being the minimum anchor length (15 by default). The seed distance ${d}_{{ji}}=\max \left\{\left|{y}_{i}-{y}_{j}\right|,\,\left|{x}_{i}-{x}_{j}\right|\right\}$ and *D* is the maximum seed distance (1,000 by default). The seed gap ${g}_{{ji}}=\left|\left|{\;y}_{i}-{y}_{j}\right|-\left|{x}_{i}-{x}_{j}\right|\right|$ and *G* is the maximum gap (50 by default). The gap penalty is calculated as $\gamma \left(g\right)=0.1\times g+0.5\,{\log }_{2}(g)$. Additionally, a filter is applied to avoid a nonmonotonic increase or decrease in coordinates. For cases where there is only one anchor, the length of the anchor must be no less than a threshold *P*′ (17 by default).

### Alignment

In contrast to the case of minimizers or closed syncmers^39^, seeds in LexicMap do not have a small window guarantee. Hence, a pseudoalignment algorithm is further used to find similar regions from the chained region, extended by 1 kb in the upstream and downstream directions. The *k*-mers (*k* = 31) on both strands of the query sequence are stored in a prefix tree and *k*-mers of a target region are queried in the prefix tree to return matches of ${{l}_{i}}^{{\prime} }\,$ (${11\le {l}_{i}}^{{\prime} }\le 31$) in the extended chained region. After removing duplicated anchors as above, the anchors are chained with the score function:

2 $$f{\,\prime} \left(i\right)=\max\left\{\mathop{\max}\limits_{\begin{array}{c}i> j\ge 1\\ {x}_{i}-G{\prime}<{x}_{j}\le {x}_{i}\\{y}_{i}-G{\prime}< {y}_{j}\le {y}_{i}\end{array}}\left\{\;f{\;\prime}\left(j\right)+{l{\prime}}_{i}-{g{\prime} }_{{ji}}\right\},{l{\prime} }_{i}\right\}$$

where $G{\prime}$ is the maximum gap (20 by default) and seed distance ${g{\prime} }_{{ji}}=\left|\left|{y}_{i}-{y}_{j}\right|-\left|{x}_{i}-{x}_{j}\right|\right|$. The chaining is banded (100 bp or 50 anchors by default). Furthermore, chained regions across the interval regions between contigs are split. Next, regions are further extended in both ends, using a similar pseudoalignment algorithm based on 2-mer matches.

Similar regions between the reference and query sequences are further aligned with a reimplemented wavefront alignment algorithm (https://github.com/shenwei356/wfa; version 0.4.0), with a gap-affine penalty (match: 0, mismatch: 4, gap opening: 6 and gap extension: 2) and the adaptive reduction heuristic (minimum wavefront length: 10 and maximum distance: 50). Each alignment’s bit score and expect value (*e* value) are computed with Karlin–Altschul parameter^40^ values for substitution scores of 2 and −3 (gap opening cost: 5, gap extension cost: 2, lambda: 0.625 and *K*: 0.41) from BLASTn’s source code.

### Tools, reference genome datasets and query sequences

LexicMap version 0.7.0, BLAST++ version 2.15.0, MMseqs2 version 16.747c6, Minimap2 version 2.28-r1209, Ropebwt3 version 3.9-r259, COBS (iqbal-lab-org fork version 0.3.0; https://github.com/iqbal-lab-org/cobs) and Phylign (AllTheBacteria fork https://github.com/AllTheBacteria/Phylign; commit 9fc65e6) were used for testing. The GTDB r214 complete dataset with 402,538 prokaryotic assemblies was downloaded with genome_updater (https://github.com/pirovc/genome_updater; version 0.6.3). The GenBank + RefSeq dataset with 2,340,672 prokaryotic assemblies was downloaded with genome_updater on February 15, 2024. The AllTheBacteria version 0.2 high-quality genome dataset with 1,858,610 bacteria genomes and the Phylign index were downloaded from GitHub (https://github.com/AllTheBacteria/AllTheBacteria; archived at https://ftp.ebi.ac.uk/pub/databases/AllTheBacteria/Releases/0.2/indexes/phylign/). Four query datasets were used for evaluating alignment accuracy and performance: a preprotein translocase subunit *secY* gene CDS sequence from an *E.* *faecalis* strain (NZ_CP064374.1_cds_WP_002359350.1_906), a 16S rRNA gene *rrsB* from *E.* *coli* str. K-12 substr. MG1655 (NC_000913.3: 4166659–4168200), a plasmid from a *Serratia nevei* strain (CP115019.1) and 1,033 AMR genes from the Bacterial Antimicrobial Resistance Reference Gene Database (PRJNA313047). All files were stored on a network-attached storage server equipped with HDD disks.

### Simulating mutations

Ten bacterial genomes of common species (more details in Supplementary Table 5) with genome sizes ranging from 2.1 to 6.3 Mb were used for simulating queries with Badread version 0.4.1 and SeqKit^41^ version 2.8.2. The command ‘badread simulate --seed 1 --reference $ref --quantity 30x --length $qlen,0 --identity $ident,$ident,0 --error_model random --qscore_model ideal --glitches 0,0,0 --junk_reads 0 --random_reads 0 --chimeras 0 --start_adapter_seq ‘’ --end_adapter_seq ‘’ | seqkit seq -g -m $qlen | seqkit grep -i -s -v -p NNNNNNNNNNNNNNNNNNNN -o $ref.i$ident.q$qlen.fastq.gz’ simulated reads with a genome coverage of 30×, a mean length of 250, 500, 1,000 or 2,000 bp and mean percentage identities ranging from 80% to 100% with SNPs and indels. LexicMap, BLASTn, MMseqs2, Minimap2, Ropebwt3 and COBS were used to search simulated reads against the reference genomes. Each tool built an index for each genome and searched or aligned reads with the corresponding index. LexicMap built indices and aligned with default parameters (*m* = 20,000, *k* = 31, *P* = 15, *P*′ = 17). BLASTn built indices with default parameters and aligned reads with the default task mode ‘megablast’ using a word size of 28 (default) or 15 and the out format 6 was set to output tabular results. MMseqs2 built indices and aligned reads with the nucleotide mode and the format mode 4 was set to output tabular results. Minimap2 aligned reads with the ‘map-ont’ mode. Ropebwt3 built indices (including .fmd, .ssa and .fmd.len.gz files) with default parameters and aligned with the local alignment mode (‘ropebwt3 sw’). COBS built compact indices with *k* = 31 and a false-positive rate of 0.3 and searched reads with a *k*-mer coverage threshold of 0.33. For all reads, the source location was tracked. Alignment rate was determined by counting the proportion of reads that had an alignment position overlapping the source location (except for COBS, where it was defined as the proportion of reads detected as being present in the genome). Unless otherwise specified, all tests were performed in cluster nodes running with Intel(R) Xeon(R) Gold 6336Y CPU @ 2.40 GHz with RAM of 500 or 2,000 GB.

### Scalability testing

Seven genome sets with 1, 10, 100, 1,000, 10,000, 100,000 and 1,000,000 nonoverlapping prokaryotic genomes randomly chosen from the GenBank + RefSeq dataset were used for the scalability test. The queries consisted of the *secY* and a 16S rRNA *rrsB* gene sequences mentioned above. LexicMap, BLASTn, MMseqs2, Minimap2, Ropebwt3 and COBS built an index for each genome set and searched or aligned the queries against the corresponding index, using the options in the previous tests. LexicMap returned all possible matches by default, while other tools were explicitly set to return all matches. All tools used 48 threads. A Python script (https://github.com/shenwei356/memusg) was used to record the time and peak memory usage. Sequence alignment and searching were repeated four times on different cluster nodes over separate weeks and the average time and memory consumption were used for plotting.

### Benchmarking

For indexing, LexicMap built indices with the genome batch size 5,000 (default) for GTDB and 25,000 for the AllTheBacteria dataset. BLASTn, MMseqs2 and Minimap2 build indices with parameters as mentioned above. The Phylign index was built with default parameters, including *k* = 31 and a false-positive rate of 0.3 for the COBS index; because the index building involved three workflows with multiple steps in multiple cluster nodes, the memory and time could not be measured accurately.

The four query datasets were used for sequence alignment. LexicMap returned all possible matches by default and other tools were set to return all possible matches according to the sequence number in a database. All tools used 48 threads. The main parameters of Phylign included threads = 48, cobs_kmer_thres = 0.33, minimap_preset = ‘asm20’, nb_best_hits = 5,000,000 and max_ram_gb = 100. For the cluster mode, the maximum number of Slurm jobs was set to 100.

The sequence alignment results from the four tools were divided into three categories according to query coverage and percentage identity and the genome numbers of each category were counted for comparison. The metrics of Minimap2 and Phylign were computed by sam2tsv.py (https://gist.github.com/apcamargo/2b7ca3032c1e80333adc1e54f47a0966). Alignments of high similarity were those with a query coverage of ≥90% (genes) or 70% (plasmids) and percentage identity of ≥90%. Alignments of low similarity are those with a query coverage of <50% (genes) or 30% (plasmid) or percentage identity of <80%. The remaining alignments were marked as medium similarity.

### Reporting summary

Further information on research design is available in the Nature Portfolio Reporting Summary linked to this article.

## Online content

Any methods, additional references, Nature Portfolio reporting summaries, source data, extended data, supplementary information, acknowledgements, peer review information; details of author contributions and competing interests; and statements of data and code availability are available at 10.1038/s41587-025-02812-8.

## Supplementary information

Supplementary Information Supplementary Tables 1–5 and Figs. 1–7.

Reporting Summary

Supplementary Table Data for Figs. 3 and 4a,b.

## Data Availability

All analyses were conducted with data public genome databases: the GTDB r214 complete dataset, GenBank + RefSeq dataset (downloaded on February 15, 2024) and AllTheBacteria version 0.2.

## Extended data

**Extended Data Table 1:** Alignment performance benchmarks on AllTheBacteria high-quality dataset

Query (length)	Tool	Hits (total)	Hits (high)	Hits (medi)	Hits (low)	Time	RAM
A rare gene	LexicMap	38,062	7,935	18	30,109	112 s	3.8 GB
1,299 bp	Phylign_local	7,937	7,935	1	1	1,848 s	77.6 GB
	Phylign_cluster	7,937	7,935	1	1	1,713 s	/
A 16S rRNA gene	LexicMap	1,857,974	496,867	556,951	804,156	1,552 s	14.8 GB
1,542 bp	Phylign_local	1,017,766	483,054	434,105	100,607	7,833 s	77.0 GB
	Phylign_cluster	1,017,766	483,054	434,105	100,607	5,141 s	/
A plasmid	LexicMap	485,295	25	9,201	476,069	1,891 s	15.2 GB
52,830 bp	Phylign_local	46,822	27	9,832	36,963	2,853 s	82.6 GB
	Phylign_cluster	46,822	27	9,832	36,963	2,374 s	/
1033 AMR genes	LexicMap	25,563,227	6,693,084	3,814,828	15,055,315	44,231 s	17.7 GB
1 kb (median)	Phylign_local	11,742,865	5,796,412	2,871,952	3,074,501	9,488 s	85.9 GB
	Phylign_cluster	11,742,865	5,796,412	2,871,952	3,074,501	8,029 s	/

Hits stand for genome hits. Hits (high), hits (medium), and hits (low) mean the number of genomes with high-, medium-, and low-similarity matches (details in Methods), respectively. Alignment memory can not be accurately measured for Phylign running in multiple cluster nodes.

**Extended Data Table 2:** Alignment performance of LexicMap on GenBank+RefSeq dataset

Query	Query length	Hits (total)	Hits (high)	Hits (medi)	Hits (low)	Time	RAM
A rare gene	1,299 bp	41,718	11,746	115	29,857	3m:06s	4.0 GB
A 16S rRNA gene	1,542 bp	1,955,167	245,884	501,691	1,207,592	32m:59s	11.1 GB
A plasmid	52,830 bp	560,330	96	15,370	544,864	52m:22s	14.5 GB
1033 AMR genes	1 kb (median)	30,967,882	7,636,386	4,858,063	18,473,433	15h:52m:08s	24.9 GB

Sequence identifiers are available in Methods. Hits stand for genome hits. Hits (high), hits (medium), and hits (low) mean the number of genomes with high-, medium-, and low-similarity matches (details in Methods), respectively.

## Code availability

Full details on how to reproduce all analyses, along with lists of accessions used, can be found on GitHub (https://github.com/shenwei356/lexicmap-benchmark) and Zenodo (10.5281/zenodo.15628530)^42^. LexicMap is an open-source standalone tool implemented in Go under the MIT license (https://github.com/shenwei356/LexicMap), with freely available statically linked executable binary files for common operating systems and CPU types. The source code is also archived on Zenodo (10.5281/zenodo.15197523)^43^. Two main subcommands ‘index’ and ‘search’ are used to create an index and perform alignment, respectively, and several utility subcommands are available for interpreting the index data and extracting indexed sequences.
