# Supplementary material for: Efficient sequence alignment against millions of prokaryotic genomes with LexicMap
Source: Nat Biotechnol. 2025 Sep 10;44(8):1383–90. doi: 10.1038/s41587-025-02812-8 (PMC13461342; doi:10.1038/s41587-025-02812-8)
Supplement: Supplementary file 1 [file 41587_2025_2812_MOESM1_ESM.pdf]

nature biotechnology

Supplementary information

https://doi.org/10.1038/s41587-025-02812-8

Efficient sequence alignment against millions of prokaryotic genomes with LexicMap

In the format provided by the authors and unedited

Supplementary Information for "Efficient sequence alignment against millions of prokaryotic genomes with LexicMap"

Wei Shen[1,2,*], John A. Lees[2], Zamin Iqbal[2,3,*]

[1] Department of Infectious Diseases, Key Laboratory of Molecular Biology for Infectious Diseases (Ministry of Education), Institute for Viral Hepatitis, The Second Affiliated Hospital, Chongqing Medical University, Chongqing, China

[2] European Molecular Biology Laboratory - European Bioinformatics Institute, Hinxton, UK

[3] Milner Centre for Evolution, University of Bath, UK

[*]Correspondence: shenwei356@cqmu.edu.cn (W.S.) and zi245@bath.ac.uk (Z.I.)

Supplementary Table 1. Index sizes and indexing performances with different numbers of probes. The reference genomes were 402,538 prokaryotic assemblies from GTDB r214 complete dataset. LexicMap `index` command used default parameters except for the number of probes. The tests were performed on a server with Intel(R) Xeon(R) Gold 6248 CPU @ 2.50GHz and all files are stored on HDD disks.

Probes	Index size	Indexing time	Indexing memory
10,000	955 GB	10 h 11 m	69.77 GB
20,000	972 GB	12 h 11 m	77.03 GB
40,000	1,029 GB	19 h 50 m	86.59 GB

Supplementary Table 2. Search performance on GTDB r214 complete dataset on three queries with different numbers of probes. We used 3 queries. A gene *secY* (termed a rare gene), a 16s rRNA gene *rrsB*, and a plasmid (accessions are available in Methods). We report the number of genomes with alignments (genome hits), and how many of these matched specific criteria. QcovHSP is the query coverage per High-scoring Segment Pair (HSP). Pident is the percentage identity. LexicMap `search` command used default parameters. The computation environment was the same as Supplementary Table 1.

Query (length)	Probes	Search time	Search memory	Genome hits	Genome hits (qcovHSP≥70)	Genome hits (qcovHSP≥70 and pident≥80)
**A rare gene**	10,000	67 s	1.77 GB	6,100	6,068	2,355
1,299 bp	20,000	70 s	2.42 GB	6,230	6,197	2,356
	40,000	64 s	3.96 GB	5,882	5,848	2,356
**A 16S rRNA gene**	10,000	749 s	3.35 GB	303,037	227,095	117,104
1,542 bp	20,000	723 s	4.31 GB	303,664	227,275	117,105
	40,000	714 s	6.14 GB	305,360	227,264	117,107
**A plasmid**	10,000	483 s	4.31 GB	60,800	755	755
52,830 bp	20,000	627 s	5.86 GB	63,517	1,191	1,191
	40,000	732 s	7.52 GB	64,970	1,191	1,191

Supplementary Table 3. Searching performance on three queries with different paired minimum anchor length thresholds. The index was built with 20,000 probes from 402,538 prokaryotic assemblies from GTDB r214 complete dataset. LexicMap `search` command used default parameters except for the paired minimum anchor length thresholds (*P* and *P'*, see methods). The computation environment was the same as Supplementary Table 1.

Query (length)	Minimum anchor length pair	Searching time	Searching memory	Genome Hits	Genome hits (qcovHSP≥70)	Genome hits (qcovHSP≥70 and pident≥80)
**A rare gene**	15-15	330 s	2.19 GB	6,505	6,269	2,356
1,299 bp	15-17	67 s	2.11 GB	6,230	6,197	2,356
	17-17	66 s	1.97 GB	6,211	6,078	2,356
	17-19	34 s	1.88 GB	6,111	6,081	2,356
	19-19	34 s	1.93 GB	6,084	6,055	2,355
	19-21	29 s	1.90 GB	5,510	5,487	2,352
**A 16S rRNA gene**	15-15	821 s	4.19 GB	304,867	227,285	117,105
1,542 bp	15-17	723 s	4.29 GB	303,664	227,275	117,105
	17-17	721 s	4.18 GB	303,353	227,212	117,105
	17-19	710 s	4.11 GB	302,071	227,137	117,105
	19-19	708 s	4.11 GB	301,867	227,053	117,104
	19-21	700 s	4.04 GB	300,413	226,953	117,104
**A plasmid**	15-15	1,191 s	6.06 GB	64,328	1,191	1,191
52,830 bp	15-17	627 s	6.08 GB	63,517	1,191	1,191
	17-17	622 s	5.74 GB	63,489	1,191	1,191
	17-19	362 s	5.85 GB	62,002	1,191	1,191
	19-19	363 s	6.24 GB	61,938	1,191	1,191
	19-21	327 s	5.67 GB	60,756	1,191	1,191

Supplementary Table 4. LexicMap alignment output format details.

#	Column	Description
1	query	Query sequence ID
2	qlen	Query sequence length
3	hits	Number of subject genomes
4	sgenome	Subject genome ID
5	sseqid	Subject sequence ID
6	qcovGnm	Query coverage (percentage) per genome: $(aligned bases in the genome)/$qlen
7	cls	Nth HSP cluster in the genome
8	hsp	Nth HSP in the genome
9	qcovHSP	Query coverage (percentage) per HSP: $(aligned bases in a HSP)/$qlen
10	alenHSP	Aligned length in the current HSP
11	pident	Percentage of identical matches in the current HSP
12	gaps	Gaps in the current HSP
13	qstart	Start of alignment in query sequence
14	qend	End of alignment in query sequence
15	sstart	Start of alignment in subject sequence
16	send	End of alignment in subject sequence
17	sstr	Subject strand
18	slen	Subject sequence length
19	evalue	Expect value
20	bitscore	Bit score
21	cigar	CIGAR string of the alignment. (optional with -a/--all)
22	qseq	Aligned part of query sequence. (optional with -a/--all)
23	sseq	Aligned part of subject sequence. (optional with -a/--all)
24	align	Alignment text ("|" and " ") between qseq and sseq. (optional with -a/--all)

Supplementary Table 5. Details of 10 bacterial genomes for the alignment rate comparison.

Assembly	TaxID	Species	Contigs	Genome size
GCF_000005845.2	562	*Escherichia coli*	1	4,641,652
GCF_000240185.1	573	*Klebsiella pneumoniae*	7	5,682,322
GCF_000013425.1	1280	*Staphylococcus aureus*	1	2,821,361
GCF_000006945.2	28901	*Salmonella enterica*	2	4,951,383
GCF_001457635.1	1313	*Streptococcus pneumoniae*	1	2,110,968
GCF_000195955.2	1773	*Mycobacterium tuberculosis*	1	4,411,532
GCF_000006765.1	287	*Pseudomonas aeruginosa*	1	6,264,404
GCF_008632635.1	470	*Acinetobacter baumannii*	2	3,980,230
GCF_018885085.1	1496	*Clostridioides difficile*	2	4,095,894
GCF_022869705.1	1351	*Enterococcus faecalis*	1	2,866,948

Supplementary Table 6. Data of Fig.3

Supplementary Table 7. Data of Fig.4a

Supplementary Table 8. Data of Fig.4b

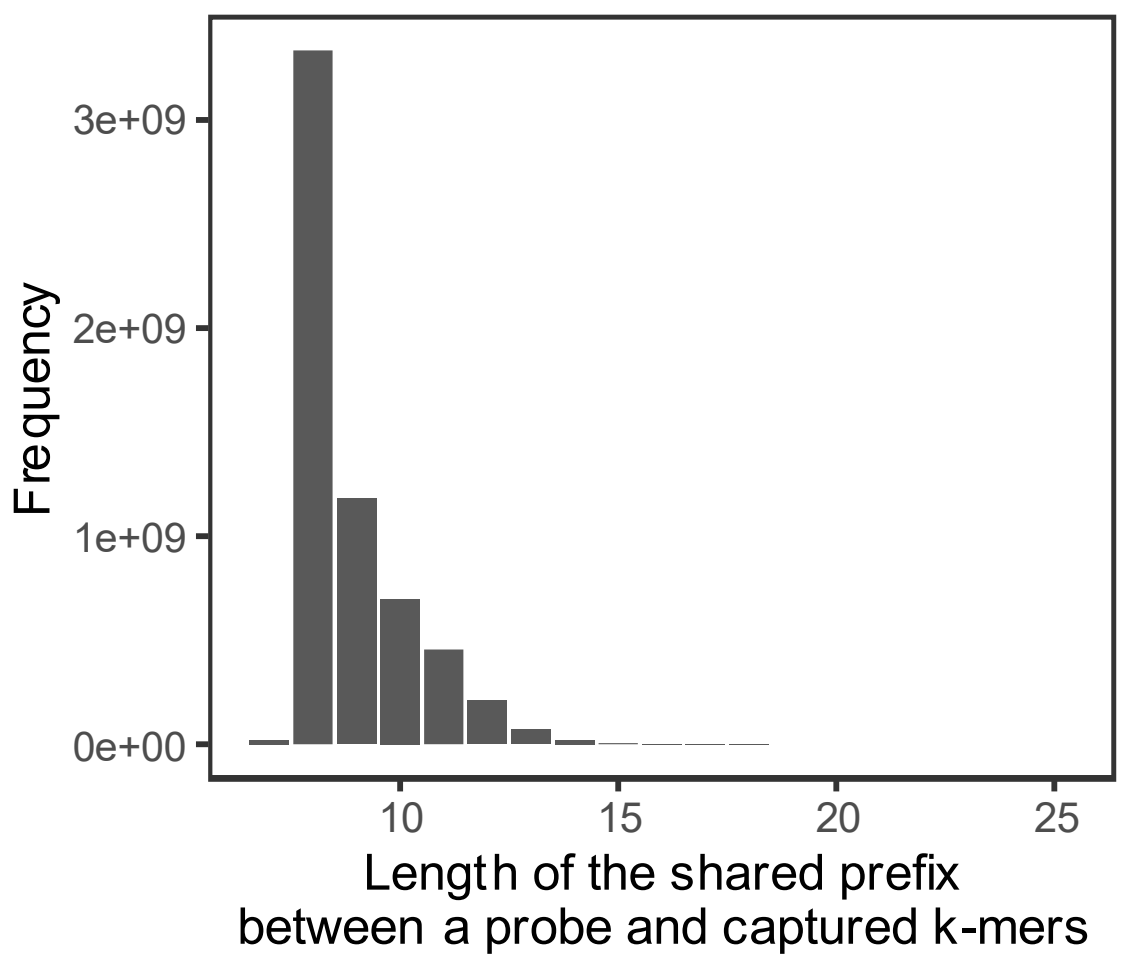

Supplementary Figure 1. Histogram of shared prefix lengths between probes and the captured *k*-mers. Using a LexicMap index of GTDB r214 representative genomes (n=85,205), we show the lengths of shared prefixes between the 20,000 fixed probes and the captured *k*-mers.

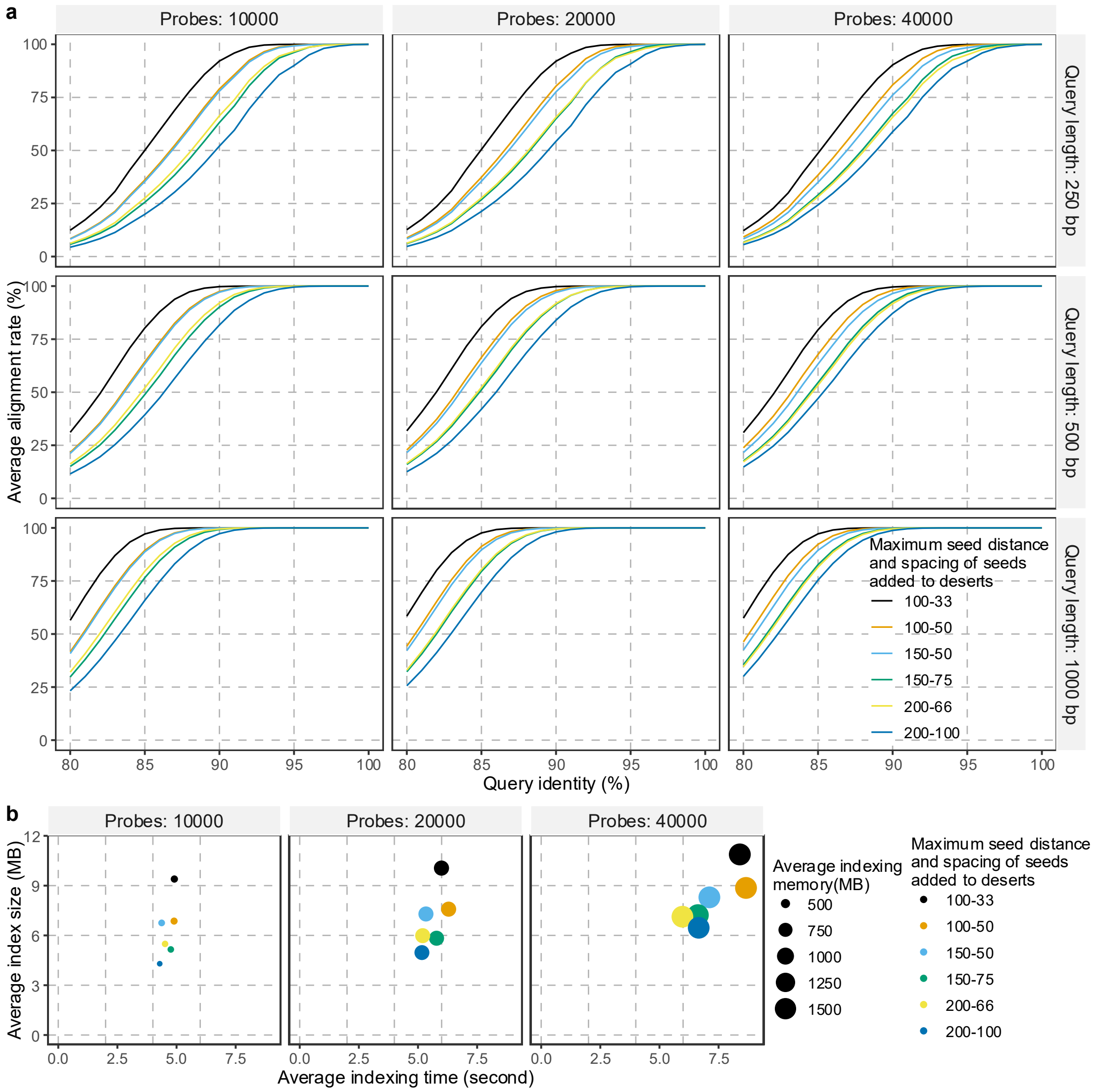

Supplementary Figure 2. Performance of LexicMap with different numbers of probes and seed densities. a) Robustness to sequence divergence. **b)** Average index sizes of single genomes and indexing performances. The same 10 reference genomes and simulated queries are the same as those in Fig.3.

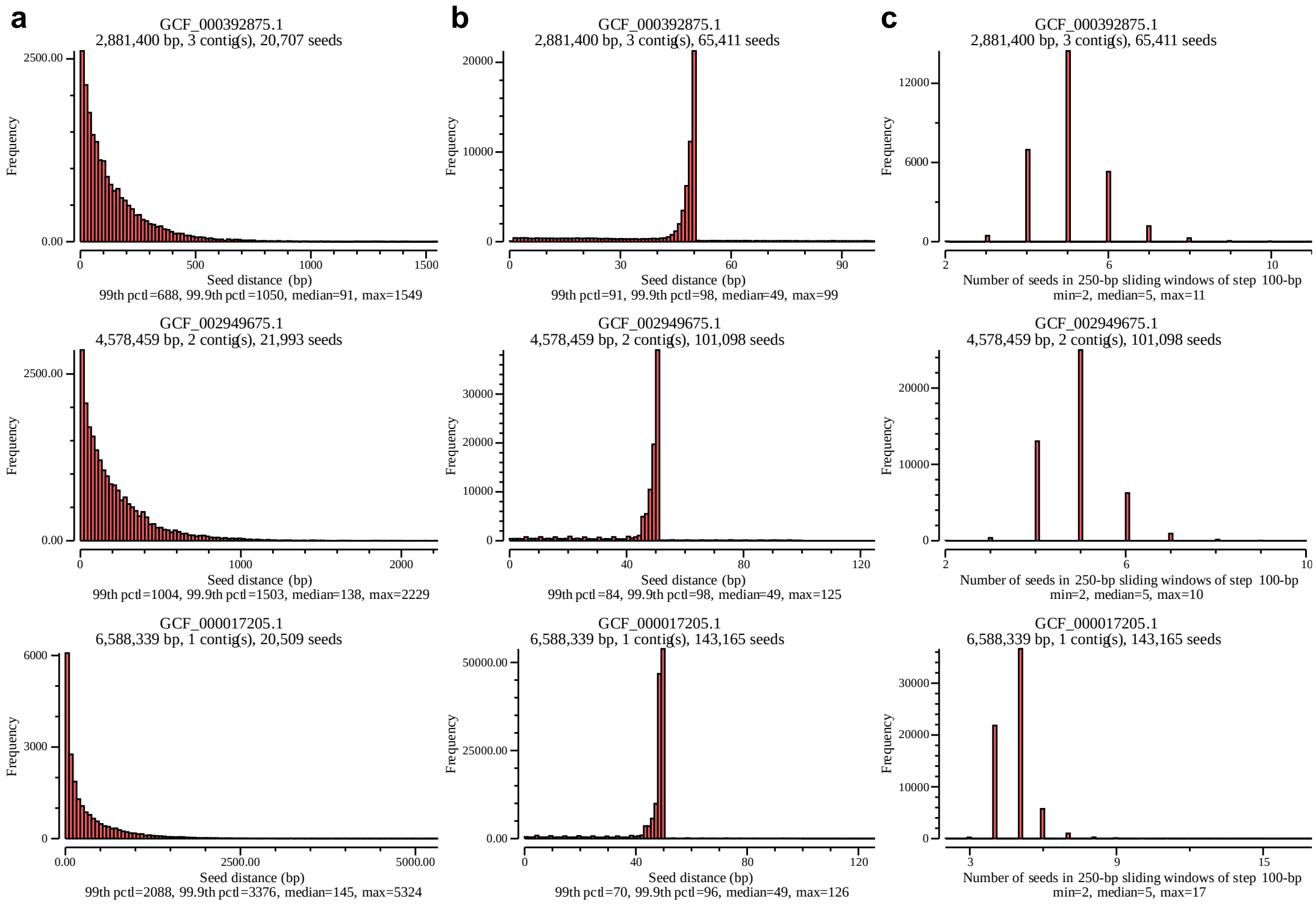

Supplementary Figure 3. Histograms of seed distance and seed number in sliding windows. Three genomes of different genome sizes are used for illustration. **a)** Histograms of seed distance before filling seed deserts. **b)** Histograms of seed distance after filling deserts. Note that in assemblies GCF_002949675.1 and GCF_000017205.1, the two maximum seed distances larger than 100 bp resulted from low-complexity regions. **c)** Histograms of seed number in 250-bp sliding windows after filling deserts.

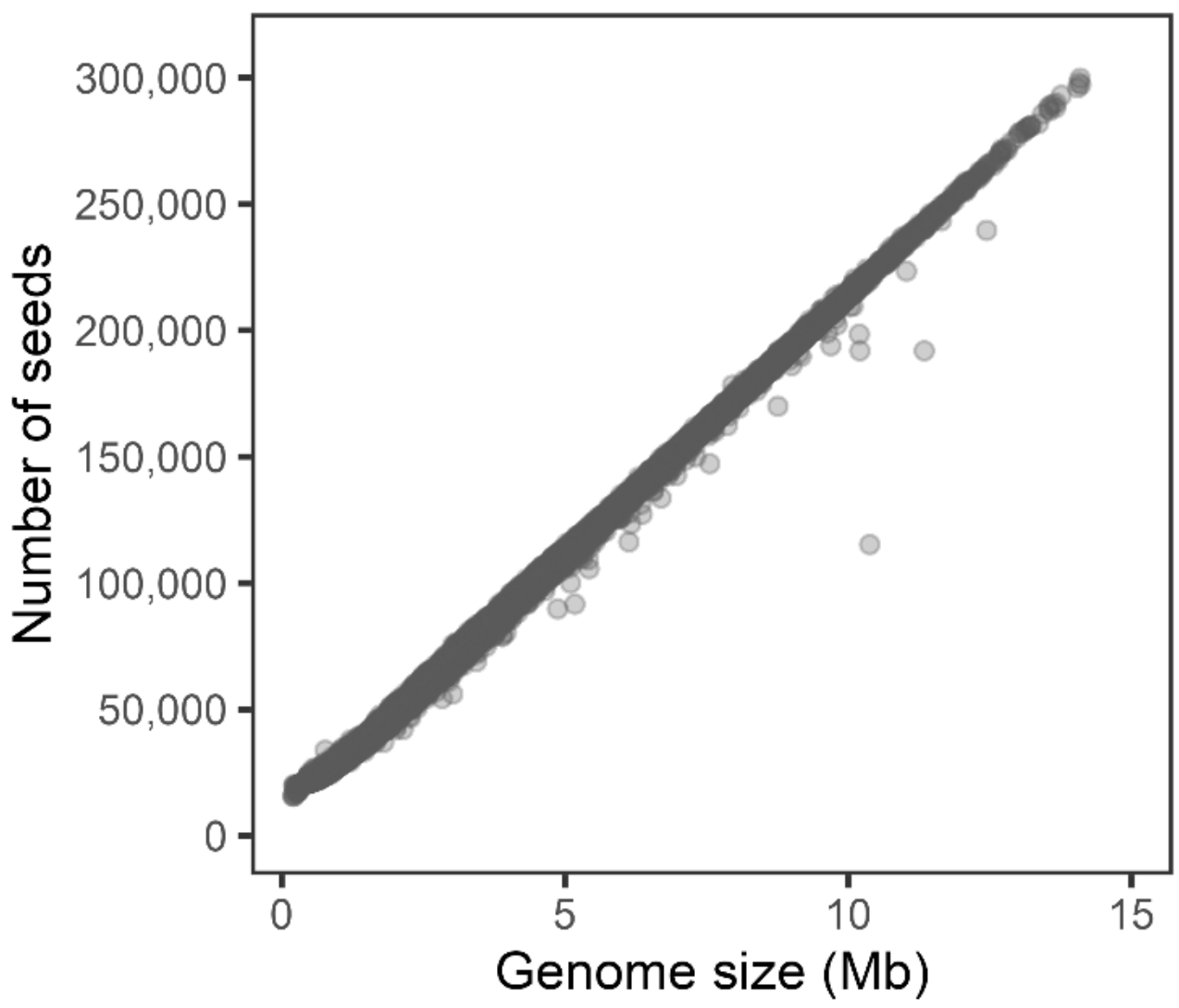

Supplementary Figure 4. Number of seeds vs genome size. A LexicMap index was built from 85,205 GTDB r214 representative genomes with default parameters and "--save-seed-pos". The plot shows the number of seeds in each genome, versus the genome size (number of bases in Megabases).

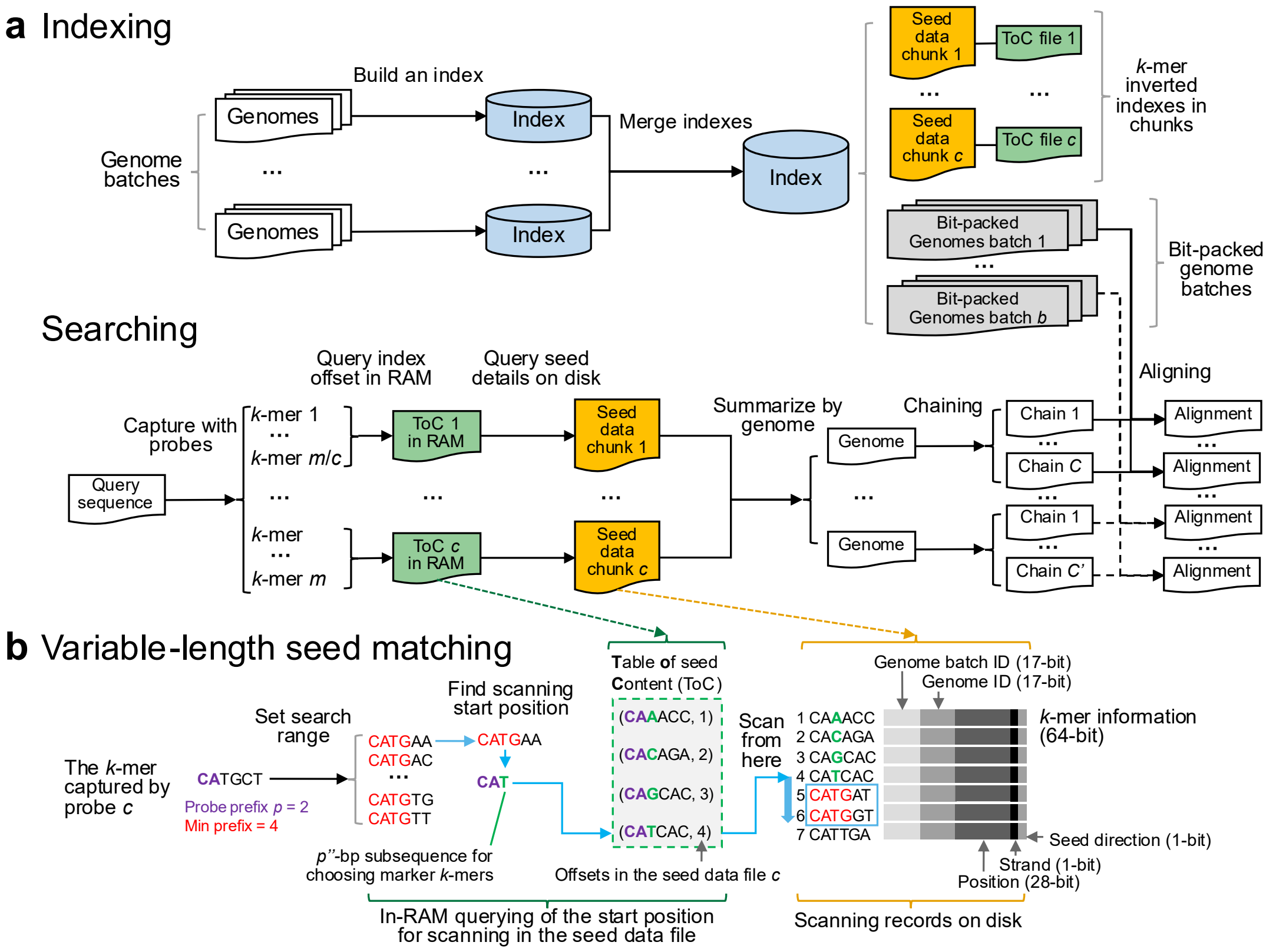

Supplementary Figure 5. Architecture of LexicMap. a) Indexing and searching workflow. **b)** Variable-length seed matching. For example, searching with CATGCT for seeds (with $p = 2$, p'' = 1) that have at least 4 bp common prefixes is equivalent to finding seeds in the range of CATGAA to CATGTT. The process starts by extracting the p''-bp subsequence from CATGAA (in this case, T) to locate the marker *k*-mer (e.g., CATCAC) with the same p''-bp subsequence in the same region. The offset information of this marker *k*-mer is then used as the starting point for scanning seeds within the *k*-mer range. Note that in (b), only prefix matching is illustrated, and suffix matching is not shown for simplicity.

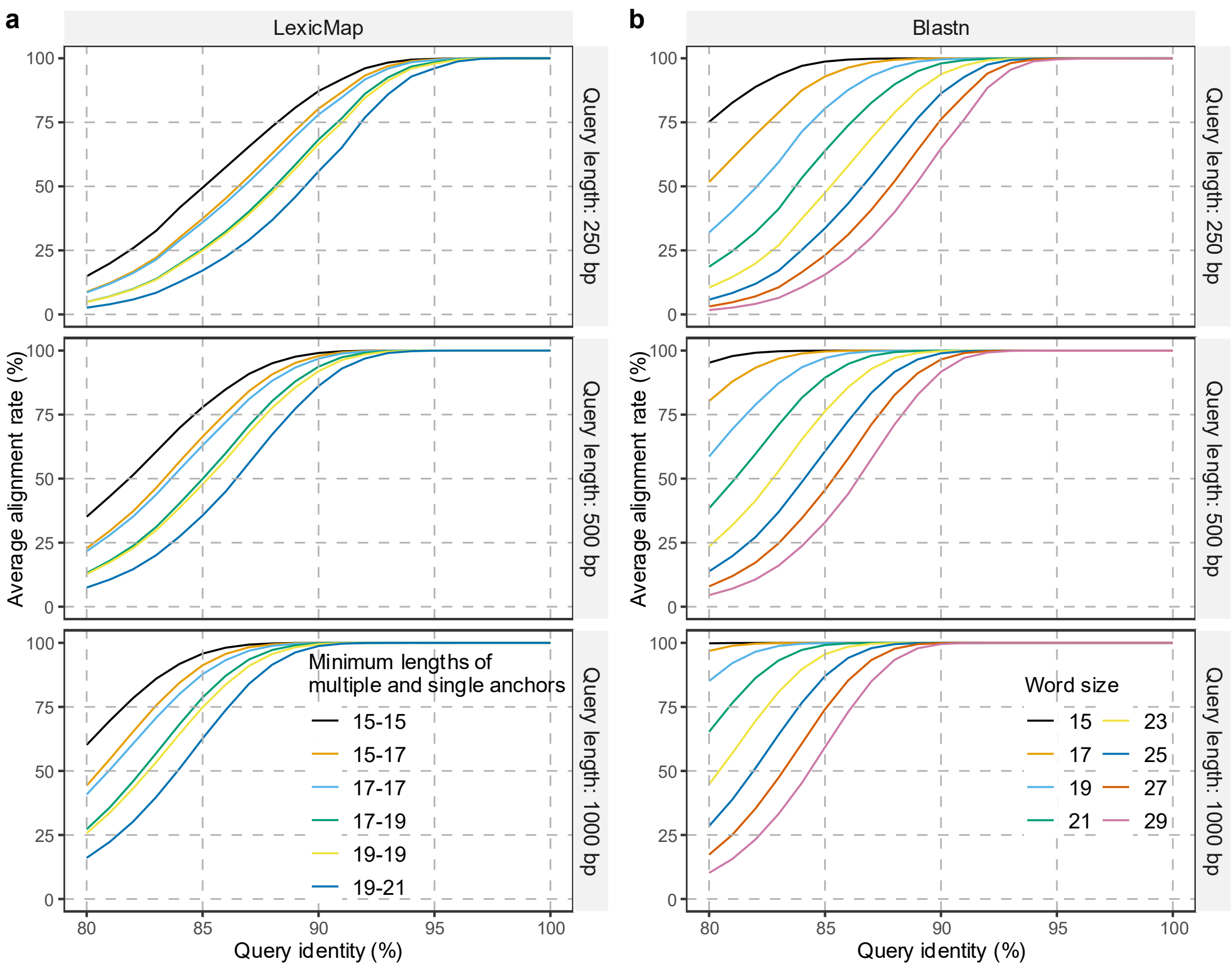

Supplementary Figure 6. Robustness of LexicMap and Blastn to sequence divergence. LexicMap uses different paired minimum anchor length thresholds (*P* and *P'*, see methods), and Blastn uses a range of word sizes. The 10 reference genomes and simulated queries are the same as those in Fig.3.

```
Query = GCF_003697165.2_r40
Length = 186

[Subject genome #1/2] = GCF_002950215.1 Shigella flexneri
Query coverage per genome = 93.548%

>NZ_CP026788.1
Length = 4659463

 HSP cluster #1, HSP #1
 Score = 279 bits, Expect = 9.66e-75
 Query coverage per seq = 93.548%, Aligned length = 177, Identities = 88.701%, Gaps = 6
 Query range = 13-186, Subject range = 1124816-1124989, Strand = Plus/Plus

Query  13       CGGAAACTGAAACA-CCAGATTCTACGATGATTATGATGATTTA-TGCTTTCTTTACTAA  70
                |||||||||||||| |||||||||| | |||||||||||||||| |||||||||| ||||
Sbjct  1124816  CGGAAACTGAAACAACCAGATTCTATGTTGATTATGATGATTTAATGCTTTCTTTGCTAA  1124875

Query  71       AAAGTAAGCGGCCAAAAAAATGAT-AACACCTGTAATGAGTATCAGAAAAGACACGGTAA  129
                ||    |||||||||||||||||| ||||||||||||||||||||||||||||||||||
Sbjct  1124876  AA--GCAGCGGCCAAAAAAATGATTAACACCTGTAATGAGTATCAGAAAAGACACGGTAA  1124933

Query  130      GAAAACACTCTTTTGGATACCTAGAGTCTGATAAGCGATTATTCTCTCTATGTTACT  186
                 || |||||||||    |||||  |||||||||||||||||||||||| |||| |||
Sbjct  1124934  AAAGACACTCTTTGAAGTACCTGAAGTCTGATAAGCGATTATTCTCTCCATGT-ACT  1124989
```

Supplementary Figure 7. Blast-style pairwise alignment output format in LexicMap. Note that, in addition to data that BLAST outputs, LexicMap also provides subject genome information (here *Shigella flexneri*) and query coverage per genome.
