# Supplementary material for: Efficient sequence alignment against millions of prokaryotic genomes with LexicMap
Source: Nat Biotechnol. 2025 Sep 10;44(8):1383–90. doi: 10.1038/s41587-025-02812-8 (PMC13461342; doi:10.1038/s41587-025-02812-8)
Supplement: Supplementary file 2 [file 41587_2025_2812_MOESM2_ESM.pdf]

nature portfolio

Corresponding author(s): Zamin Iqbal

Last updated by author(s): Aug 12, 2025

Reporting Summary

Nature Portfolio wishes to improve the reproducibility of the work that we publish. This form provides structure for consistency and transparency in reporting. For further information on Nature Portfolio policies, see our Editorial Policies and the Editorial Policy Checklist.

Statistics

For all statistical analyses, confirm that the following items are present in the figure legend, table legend, main text, or Methods section.

n/a	Confirmed	
☐	☒	The exact sample size (*n*) for each experimental group/condition, given as a discrete number and unit of measurement
☒	☐	A statement on whether measurements were taken from distinct samples or whether the same sample was measured repeatedly
☒	☐	The statistical test(s) used AND whether they are one- or two-sided<br>*Only common tests should be described solely by name; describe more complex techniques in the Methods section.*
☒	☐	A description of all covariates tested
☒	☐	A description of any assumptions or corrections, such as tests of normality and adjustment for multiple comparisons
☒	☐	A full description of the statistical parameters including central tendency (e.g. means) or other basic estimates (e.g. regression coefficient) AND variation (e.g. standard deviation) or associated estimates of uncertainty (e.g. confidence intervals)
☒	☐	For null hypothesis testing, the test statistic (e.g. *F*, *t*, *r*) with confidence intervals, effect sizes, degrees of freedom and *P* value noted<br>*Give P values as exact values whenever suitable.*
☒	☐	For Bayesian analysis, information on the choice of priors and Markov chain Monte Carlo settings
☒	☐	For hierarchical and complex designs, identification of the appropriate level for tests and full reporting of outcomes
☒	☐	Estimates of effect sizes (e.g. Cohen's *d*, Pearson's *r*), indicating how they were calculated

Our web collection on statistics for biologists contains articles on many of the points above.

Software and code

Policy information about availability of computer code

Data collection	All analyses were done with data public genome databases; specifically GTDB r214 complete dataset, GenBank+RefSeq dataset, downloaded on February 15, 2024, and AllTheBacteria v0.2.
Data analysis	LexicMap is an open-source standalone tool implemented in Go under the MIT license at https://github.com/shenwei356/LexicMap, with freely available statically linked executable binary files for common operating systems and CPU types. The source code is also archived at https://doi.org/10.5281/zenodo.15197523. Full details on how to reproduce all analyses, along with lists of accessions used, can be found in this GitHub repository https://github.com/shenwei356/lexicmap-benchmark, and also in this Zenodo archive: https://doi.org/10.5281/zenodo.15628530.

For manuscripts utilizing custom algorithms or software that are central to the research but not yet described in published literature, software must be made available to editors and reviewers. We strongly encourage code deposition in a community repository (e.g. GitHub). See the Nature Portfolio guidelines for submitting code & software for further information.

Data

Policy information about availability of data

All manuscripts must include a data availability statement. This statement should provide the following information, where applicable:

- Accession codes, unique identifiers, or web links for publicly available datasets
- A description of any restrictions on data availability
- For clinical datasets or third party data, please ensure that the statement adheres to our policy

All analyses were done with data public genome databases; specifically GTDB r214 complete dataset, GenBank+RefSeq dataset, downloaded on February 15, 2024, and AllTheBacteria v0.2. Full details on how to reproduce all analyses, along with lists of accessions used, can be found in this GitHub repository https://github.com/shenwei356/lexicmap-benchmark, and also in this Zenodo archive: https://doi.org/10.5281/zenodo.15628530 .

Research involving human participants, their data, or biological material

Policy information about studies with human participants or human data. See also policy information about sex, gender (identity/presentation), and sexual orientation and race, ethnicity and racism.

Reporting on sex and gender	N/A
Reporting on race, ethnicity, or other socially relevant groupings	N/A
Population characteristics	N/A
Recruitment	N/A
Ethics oversight	N/A

Note that full information on the approval of the study protocol must also be provided in the manuscript.

Field-specific reporting

Please select the one below that is the best fit for your research. If you are not sure, read the appropriate sections before making your selection.

☒ Life sciences ☐ Behavioural & social sciences ☐ Ecological, evolutionary & environmental sciences

For a reference copy of the document with all sections, see nature.com/documents/nr-reporting-summary-flat.pdf

Life sciences study design

All studies must disclose on these points even when the disclosure is negative.

Sample size	Our study is about building indexes of large datasets. We needed to test two things. First: ability of the method to detect sequence that was evolutionarily diverged from corresponding sequence in a database. To do this, we simulated data of progressively increasing genetic divergence from a set of bacterial species, and then benchmarked (our tool and others) for the ability to match them back to their source.. This was the most precise way to measure this. Secondly, we wanted to measure ability to scale as database size increased. We therefore created databases of size ranging from 1 genome to 1 million, taking genomes from GenBank and RefSeq. This allowed us to creat realistic representations of the actual databases we want the tool to work on. Finally we ran on the entirety of GenBank+RefSeq, and AllTheBacterial (2.4 million genomes and 1.8 million genomes) - i.e. all bacterial sequence data to date. This therefore is the precise data needed to measure if the method will scale to global sequenced microbial data. Reproducibility was ensured in two ways. First, all code was versioned and made available. Second, the scalability measurements were repeated, and indeed we compared the full scale up results from two different datasets of around 2 million genomes (Refseq+Genbank, and AllTheBacteria). We also confirmed the results were not sure to bias towards highly sampled species, by testing with both common and rare species. There was no notion of different groups being created or compared, so blinding of authors was not relevant.
Data exclusions	No exclusions
Replication	We test on multiple databases
Randomization	N/A
Blinding	N/A

Reporting for specific materials, systems and methods

We require information from authors about some types of materials, experimental systems and methods used in many studies. Here, indicate whether each material, system or method listed is relevant to your study. If you are not sure if a list item applies to your research, read the appropriate section before selecting a response.

Materials & experimental systems

n/a	Involved in the study
☒	☐ Antibodies
☒	☐ Eukaryotic cell lines
☒	☐ Palaeontology and archaeology
☒	☐ Animals and other organisms
☒	☐ Clinical data
☒	☐ Dual use research of concern
☒	☐ Plants

Methods

n/a	Involved in the study
☒	☐ ChIP-seq
☒	☐ Flow cytometry
☒	☐ MRI-based neuroimaging

Plants

Seed stocks	N/A
Novel plant genotypes	N/A
Authentication	N/A
